# Anti-*Wolbachia* drug discovery and development: safe macrofilaricides for onchocerciasis and lymphatic filariasis

**DOI:** 10.1017/S0031182013001108

**Published:** 2013-07-18

**Authors:** MARK J. TAYLOR, ACHIM HOERAUF, SIMON TOWNSON, BARTON E. SLATKO, STEPHEN A. WARD

**Affiliations:** 1 Liverpool School of Tropical Medicine, Pembroke Place, Liverpool, L3 5QA, UK; 2 Institute for Medical Microbiology, Immunology and Parasitology, University Hospital Bonn, Sigmund-Freud-Strasse 25, 53105 Bonn, Germany; 3 Tropical Parasitic Diseases Unit, Northwick Park Institute for Medical Research, Watford Road, Harrow, Middlesex HA1 3UJ, UK; 4 New England Biolabs, Inc., 240 County Road, Ipswich, MA 01938, USA

**Keywords:** *Wolbachia*, onchocerciasis, lymphatic filariasis, drug discovery, macrofilaricide

## Abstract

Anti-*Wolbachia* therapy delivers safe macrofilaricidal activity with superior therapeutic outcomes compared to all standard anti-filarial treatments, with the added benefit of substantial improvements in clinical pathology. These outcomes can be achieved, in principle, with existing registered drugs, e.g. doxycycline, that are affordable, available to endemic communities and have well known, albeit population-limiting, safety profiles. The key barriers to using doxycycline as an mass drug administration (MDA) strategy for widespread community-based control are the logistics of a relatively lengthy course of treatment (4–6 weeks) and contraindications in children under eight years and pregnancy. Therefore, the primary goal of the anti-*Wolbachia* (A·WOL) consortium is to find drugs and regimens that reduce the period of treatment from weeks to days (7 days or less), and to find drugs which would be safe in excluded target populations (pregnancy and children). A secondary goal is to refine regimens of existing antibiotics suitable for a more restricted use, prior to the availability of a regimen that is compatible with MDA usage. For example, for use in the event of the emergence of drug-resistance, in individuals with high loiasis co-infection and at risk of severe adverse events (SAE) to ivermectin, or in post-MDA ‘endgame scenarios’, where test and treat strategies become more cost effective and deliverable.

## INTRODUCTION

Filariasis continues to inflict serious public health problems throughout tropical communities. The major disease-causing species include those responsible for lymphatic filariasis (LF), *Wuchereria bancrofti* and *Brugia malayi*, and onchocerciasis, *Onchocerca volvulus*, which together infect more than 150 million people, ranking filariasis as one of the leading causes of global morbidity (Taylor *et al.* 2010).

Global programmes for control and elimination have been developed to provide sustained delivery of drugs to affected communities in order to interrupt transmission of disease and ultimately eliminate this public health burden (Amazigo, 2008; Sauerbrey, 2008; WHO, 2010). Currently used drugs, diethylcarbamazine/albendazole or ivermectin/albendazole for LF and ivermectin (IVM) for onchocerciasis, principally target the microfilarial stage of the parasites and so require sustained and prolonged delivery with high treatment coverage to endemic communities in order to break the transmission cycle of the long-lived adult worms (*O. volvulus*, 10–14 years; *W. bancrofti*/*B. malayi*, 5–8 years). The impressive impact of these MDA programmes on public health is well documented (Chu *et al.* 2010; Coffeng *et al.* 2013), yet important challenges remain as these programmes translate from control to elimination goals (Bockarie and Deb, 2010; Mackenzie *et al.* 2012). Seventeen countries in hard-to-reach areas, including post-conflict countries, have still not implemented mass drug administration (MDA) against LF 12 years after the GPELF was launched. In some of these countries, interruption of transmission will not be achieved using the current strategy alone. The growing evidence for resistance to IVM (Taylor *et al.* 2009; Osei-Atweneboana *et al.* 2011) and safety constraints in areas co-endemic with *Loa loa* (Scientific Working Group on Serious Adverse Events in *Loa Loa* endemic area, 2003) has re-focused the need and urgency for new safe macrofilaricidal drugs and regimens to achieve elimination goals within existing timeframes (WHO, 2012).

Anti-*Wolbachia* therapy delivers safe macrofilaricidal activity with superior therapeutic outcomes compared to all standard anti-filarial treatments, with the added benefit of substantial improvements in clinical pathology (Taylor *et al.* 2010; Tamarozzi *et al.* 2011). These outcomes can be achieved, in principle, with existing registered drugs, e.g. doxycycline, that are affordable, available to endemic communities and have well known, albeit population-limiting, safety profiles. Anti-*Wolbachia* therapy delivers an early block in embryogenesis and gradual macrofilaricidal activity leading to a progressive and sustained elimination of microfilarial load, thus avoiding the risk of SAE from target species and those due to co-infections with *L. loa* (a species without *Wolbachia*) (Taylor *et al.* 2005). The use of doxycycline as a macrofilaricidal therapy has been established as proof-of-concept in an extensive series of field trials (reviewed in Hoerauf, 2008; Taylor *et al*. 2010), but its widespread use in community-based control is constrained by the logistics of a relatively lengthy course of treatment (4–6 weeks) and contraindications in children under eight years and pregnancy. These barriers stimulated the formation of the ‘Anti-*Wolbachia*’ (A·WOL) consortium in 2007, which was funded by the Bill & Melinda Gates Foundation to search for new drugs active against *Wolbachia* that are suitable for community-directed MDA with a secondary goal to optimize regimens of existing drugs and re-purposed registered drugs for use in more restricted target populations (http://www.a-wol.net).

## A·WOL ASSAY DEVELOPMENT

### Screening funnel

**Fig. 1.**
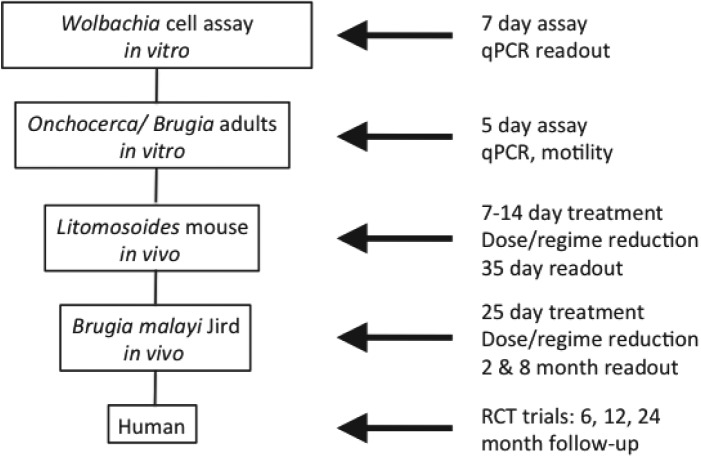
Screening funnel developed for A·WOL.

As a starting point, A·WOL developed a whole organism *Wolbachia* cell-based assay as the primary *in vitro* drug-screening tool. This validated assay, which has been adapted to automated high throughput-screening and represents a rapid, sensitive and efficient assay for screening chemical libraries, utilizes a *Wolbachia*-containing *Aedes albopictus* cell line (C6/36 Wp) (Turner *et al.* 2006), in a 96-well format, with a quantitative PCR (qPCR) read-out to quantify the *Wolbachia* 16S rRNA gene copy number following treatment (Johnston *et al.* 2010). Hits from this primary *in vitro* cell-based screening assay are selected based on their log drop depletion of *Wolbachia*, reproducibility and, if using known drugs, the target product profile (TPP) as defined by A·WOL to include oral formulation, and the safe use in children and pregnancy. These selected hits are then moved down the screening pipeline into both *in vitro* and *in vivo* nematode screening. *In vitro* nematode screening, using either adult male *Onchocerca gutturosa* (Townson *et al.* 2006) or adult *B. malayi*, is intended to verify that hits are effective against nematode *Wolbachia*. These *in vitro* screens also identify compounds that have no direct anti-nematode activity yet show significant reductions in *Wolbachia* load. For *in vivo* nematode screening, established animal models of filarial infection are utilized and include *Litomosoides sigmodontis* in mice (Hoerauf *et al*. 1999) and *B. malayi* in gerbils (Ash and Riley, 1970). For all *in vivo* models, the reduction of *Wolbachia* load following treatment is measured by qPCR. The primary *in vivo* screening model with *L. sigmodontis* allows for rapid screening of compounds and yields a visible and quantifiable phenotype of larvae with retarded growth. The secondary *in vivo* model with *B. malayi* uses a human filarial nematode and evaluates reductions in *Wolbachia* load predictive of macrofilaricidal activity, effects on female fertility and microfilarial production (Fig. 1).

### Increasing the throughput and capacity of the cell-based assay

To date, 558 000 compounds have been procured from multiple sources with ∼18 000 having completed screening in our standard cell-based assay with a qPCR read-out. In order to increase throughput and capacity of the A·WOL cell-based screen we have developed a 384-well format assay using a high content imaging system (Operetta) and optimization of growth dynamics in the C6/36 *A. albopictus* mosquito cell-line. This assay uses texture analysis of cells stained with Syto-11 as a direct measure of bacterial load and enables a shorter screening period and dramatically increases throughput and capacity. The assay has completed validation against ‘hits’ identified using the standard qPCR assay and is being used to complete diversity library screening within a 10-fold increase in throughput (Taylor *et al*. unpublished results).

## A·WOL LIBRARY SCREENING

One of the first activities was to develop a TPP for an A·WOL macrofilaricide. A·WOL worked with consultants from the pharma industry to compile four TPPs. Each TPP covered individual drug administration (IDA) or MDA for either onchocerciasis or LF. The screening campaign started with a library of registered drugs and developed to include focused libraries from pharma collaborators and large diversity-based compound libraries.

### Registered drug library

Following the validation of the primary cell-based screen, the first priority was to screen approved human drug-pharmacopeia for potential repurposing for anti-*Wolbachia* activity. Repurposing or repositioning of drugs provides a less risky route to drug discovery given that candidates will already have well-known safety and pharmacokinetic profiles, and could provide a cost- and time-effective strategy to identify a novel A·WOL therapeutic. By screening 2664 compounds from the human drug-pharmacopeia, this strategy identified 121 hits that had anti-*Wolbachia* activity; 69 of these were orally available from different diverse drug categories, with nine compounds being more potent than doxycycline. Several drugs have progressed further along the screening pipeline into *in vitro* nematode assays and *in vivo* screening models. The most advanced lead, minocycline has shown an increase in potency of 50% compared to doxycycline in the secondary *in vivo* screen and has entered efficacy trials in humans (see *Drug Regimen Refinement*, below; Taylor *et al.* unpublished results).

### Combination treatment

Combinations of registered drug-screening outputs were assayed in a doxycycline enhancer assay using sub-optimal doxycycline (50 nm) plus 21 of the registered drug hits. These outcomes were used to design an extensive series of combinations of registered A·WOL drugs. These drugs were tested in the primary *in vivo* screen in triple and double combinations, with further regimen reduction experiments to determine the shortest period of treatment. The outcome of these experiments showed that in this model, double or triple combinations of registered A·WOL drugs could reduce the period treatment to 7 days or less to deliver equivalent efficacy to a standard course of doxycycline monotherapy (Specht *et al*. unpublished results). This outcome proved that there is no biological barrier to delivering anti-*Wolbachia* therapy in shortened regimens that could meet the primary goal of an A·WOL regimen compatible with MDA.

### Focused drug libraries

Focused anti-infective libraries have been sourced from several pharmaceutical companies and include near-to-market lead candidate drugs or drug class derivatives which are selected from known and bio-informatically predicted essential gene targets (Holman *et al.* 2009, see *A·WOL Target Discovery*). Focused anti-infective library screening has, thus far, involved A·WOL *in vitro* screening of 3062 novel compounds from five chemical libraries. To date this has generated 184 diverse hit compounds, a number of which have progressed further into the screening funnel. Encouragingly, there is a good agreement between the reduction in *Wolbachia* load in the cell-based and *O. gutturosa in vitro* assays with no effect on worm motility. This suggests that the hits do not directly affect the nematode (and are, therefore, predicted to avoid direct parasite-mediated adverse events). Notably, the ability to identify hit compounds from these focused libraries which are effective at reducing *Wolbachia* load and have improved efficacy over doxycycline, is highly supportive of the long-term goal to identify A·WOL new chemical entities (NCEs).

### Lead series originating from diversity library screens

A screen of >10 000 compounds from the BioFocus library revealed compounds that showed significant anti-*Wolbachia* activity. Retesting of these hits confirmed the identity of 50 compounds as confirmed hits (hit rate 0·5%). Chemoinformatic analysis of these 50 hits has been used to identify the best hit series (consisting of ∼6 chemotypes) with the potential to enter a medicinal chemistry ‘hit to lead’ and lead optimization development phase in the A·WOL II Macrofilaricide Drug Discovery programme. We have developed a rational medicinal chemistry programme around each of the six hit series. From the top six hits we have selected three templates for hit to lead optimization with three additional back-up templates. We are currently running a head to head evaluation of the three series with the intention of identifying the most promising template for final lead optimization.

Key outcomes from A·WOL library screening include the development of a portfolio of drug discovery projects with the potential to generate at least one new anti-wolbachial chemotype for eventual deployment as a macrofilaricide monotherapy (although deployment in combination would remain an option). Evidence to date suggests that there is no biological barrier to a reduced curative dosage regimen. We have already provided proof-of-concept for this in experimental double/triple combination studies. Furthermore the life-style constraints of *Wolbachia* make acquisition of plasmid-based resistance mechanisms highly unlikely hence reducing the risk of monotherapy-driven resistance. To date several hundred ‘hits’ have been identified and confirmed from screening of focused libraries from pharma and large diversity-based libraries (Table 1).

**Table 1.** Summary of A·WOL screening campaign

Compound class	Screened *in vitro* cell assay	Hits *in vitro*	Hits confirmed using *in vitro* worm	Hits screened in 1° *in vivo*	Active (⩾doxy) in 1° *in vivo*	Hits screened in 2° *in vivo*	Active (⩾doxy) in 2° *in vivo*	Phase II trials in Ghana	Repurposing or lead optimization series
*Registered drug screening*									
Registered drugs	2664	69	✓	20	4	2	1	Minocycline	
Registered drugs	80	4		3	0				
**Total**	**2744**	**73**		**23**	**4**	**2**	**1**	**1**	
*Focused library screening*									
Tetracyclines	1084	96	✓	82	19				
Boron based	2744	179	✓	2	Ongoing	2	Ongoing		✓
Quinolones, macrolides, NRIs	312	99	✓	5	1	1	1		✓
Antibacterials, kinase inhibitors	409	62		16	6				✓
Active against Mtb	1477	22							
Novel quinolones	350	21	Ongoing						✓
Epichem-fenarimol series	50	4							
Antibacterials, anti-Mtb	1128	97							
**Total**	**7554**	**580**		**105**	**26**	**3**	**1**		
									✓
Soft-focus diversity	9946	112	Ongoing						
Natural products	2400	15							
DOS library	924	2							
Diversity	500 000	Ongoing							
Diversity	15 000	TBA							
Diversity	150 000	TBA							
**Total**	**678 270**	**129**		**nd**	**nd**	**nd**	**nd**		
**Grand total**	**688 568**	**782**		**128**	**30**	**5**	**2**	**1**	

## A·WOL TARGET DISCOVERY

### Targets of key enzymatic and metabolic pathways predicted from *Wolbachia* genomic annotation

Annotation of the *w*Bm genome suggested *Wolbachia* might provide haem, flavin adenine dinucleotide, riboflavin and nucleotides to the *B. malayi* host, which cannot synthesize these molecules *de novo* (Foster *et al.* 2005; Slatko *et al.* 2010). For example, two enzymes of the *w*Bm haem biosynthetic pathway, ALAD (aminolevulinic acid dehydratase) and FeCH (ferrochelatase), have been evaluated as candidate targets based on their low conservation to the corresponding human proteins, their distinct biochemical properties and sensitivities to inhibitors relative to the human enzymes. Inhibition of ALAD with succinyl acetone resulted in reduced worm motility *in vitro* (Wu *et al.* 2009) although FeCH is present both in *Wolbachia* and in the nematode genome through a lateral gene transfer event from an unrelated *α*-proteobacterium (Wu *et al.* 2013). Also, because ALAD is not found in the genome of *B. malayi* and is significantly different from the human orthologue, it was subjected to both aptamer- and chemical library-screening as part of the A·WOL programme.

Comparative genomic analyses and examination of metabolic pathway maps can indicate key differences between processes that are otherwise conserved between *Wolbachia* and humans leading to the identification of additional potential drug targets in *Wolbachia*. For example, the final step in glycolysis is catalysed by pyruvate kinase in humans but by a distinct alternative enzyme, pyruvate phosphate dikinase (PPDK), in *Wolbachia* (Raverdy *et al.* 2008). PPDK is not found in mammals. The *Wolbachia* PPDK enzyme has also been included in both aptamer-based and conventional library screening as part of A·WOL.

#### wALAD

The *Wolbachia* ALAD protein involved in the synthesis of haem was identified and validated as a target for drug screening. Direct screening of a small molecule library of 18 000 compounds against the enzyme assay identified 7 compounds that inhibited wALAD compared to hALAD. Three of the compounds were based on the same benzimidazole core structure. These clustered compounds also had the highest and most specific level of inhibition of wALAD. For this reason, efforts were focused on this core structure and the best inhibitor now named wALADin 1. Characterization of the inhibitory activity has identified the mode of inhibition to be a Mixed-Model Inhibition with a calculated K_I_ of 11 *μ*m. wALADin 1 was screened in the primary cell-based assay, but unfortunately showed no activity. Activity could be demonstrated against nematodes *in vitro*, although only in the 0·25–0·5 mm range (Lentz *et al.* 2013). Although this work further validated haem biosynthesis as a target, in view of the more potent and tractable hits identified through library screening, further work on wALADin 1 was suspended.

#### PPDK

The enzymatic assay for PPDK was successfully modified for a micro-titre plate format and produced acceptable Z’ values. This assay was used to screen PPDK activity against the small molecule library of 18 000 compounds. Due to the large number of hits, the threshold for our cut-off for a hit was raised from 50% inhibition to 80% inhibition. 22 compounds met or surpassed this cut-off. This list was then shortened to 7 highly active compounds that appeared to be specific for PPDK (i.e. they did not inhibit wALAD). The two best compounds were found to be non-specific, therefore further development was suspended.

#### LspA

Lipoproteins are essential structural and functional components of bacteria and those from *Wolbachia* are potent stimulators of the innate and adaptive inflammatory pathogenesis of filarial disease (Turner *et al.* 2009; Tamarozzi *et al.* 2011). The *Wolbachia* prolipoprotein signal peptidase II (LspA) was shown to be functional and the *Wolbachia* cell-based assay and adult *B. malayi* were sensitive to inhibition with a known LspA inhibitor, globomycin (Johnston *et al.* 2010) validating *LspA* as an anti-*Wolbachia* target.

### The ‘essential gene set’ of *Wolbachia*

In order to target focused library screening to drugs with predicted activity against *Wolbachia*, a bioinformatic analysis of predicted essential genes was undertaken. An essentiality score for each predicted gene of *w*Bm was determined by two separate approaches (Holman *et al.* 2009). The first method compared each gene to entries in DEG (the Database of Essential Genes), a collection of ∼5000 experimentally identified essential genes from 15 different bacterial species, to predict essential genes that are mostly conserved across the bacterial domain. The second approach used phyletic conservation across members of the order Rickettsiales, to which *Wolbachia* belongs, in order to highlight genes that are well conserved and thus likely to be essential. Conservation of genes in these rickettsial genomes that are undergoing reductive evolution underscores their importance. A ranked essentiality list was produced by each method and showed complementary and partially overlapping sets of *w*Bm genes. Many of the top-ranking genes fall into classes of genes targeted by current antibiotics and are in functional categories predicted to be essential for bacterial growth. The high essentiality prediction of such known targets validates the computational approach. The ranked lists can be further curated to prioritize candidate drug targets by filtering for genes with no similarity to human proteins for example. The druggability of the *w*Bm proteins was addressed by comparing them to known protein targets contained within the DrugBank database, a collection of ∼5000 FDA-approved small molecule drugs and compounds with details of their protein-binding partners and relevant chemical and pharmacological data. This analysis correlated well with the essential gene predictions and revealed classes of *w*Bm proteins that appear to be essential and druggable (Holman *et al.* 2009).

Screening of focused libraries generated with reference to the predicted essential gene list has delivered several lead compounds/drugs, which are undergoing further evaluation in A·WOL II Macrofilaricide Drug Discovery and A·WOL II Macrofilaricide Drug Development programmes.

### Further insight into the biological basis of *Wolbachia* symbiosis

A deeper understanding of the nature of the symbiotic relationship between *Wolbachia* and its filarial host and the consequences of *Wolbachia* depletion on the biology of the nematode has advanced in recent years (Taylor *et al.* 2012). It now appears that the dependency of the nematode–*Wolbachia* relationship is most critical during periods of high metabolic demands, such as larval development, growth and fertility, processes that coincide with periods of rapid *Wolbachia* population growth and expansion (McGarry *et al*. 2004; Taylor *et al.* 2012).

### Why does *Wolbachia* depletion induce anti-filarial activity?

Studies on the cellular consequences of symbiont elimination have provided an important insight into the cellular mechanisms at the basis of the symbiotic relationship (Landmann *et al*. 2011). Soon after antibiotic elimination of the bacteria extensive apoptosis occurs in the adult germline and in the somatic cells of the embryos, microfilariae and fourth-stage larvae (L4). Apoptosis extends to uninfected cells, suggesting an indirect provision of products from the hypodermal population is required to prevent cells from undergoing cell death. This cellular mechanism does not extend to all somatic cells, including those of the hypodermal cord cells, where the bacteria reside, although the cytoskeletal arrangement is disrupted. The pattern of apoptosis activation correlates closely with the stages most vulnerable to antibiotic depletion and provides a mechanism to account for the rapid anti-filarial effects of antibiotic treatment. Additionally apoptosis signals in host nematodes could serve as useful biomarkers of anti-*Wolbachia* activity.

### *Wolbachia* populations are regulated by autophagy and autophagy-inducing drugs deliver bactericidal activity

In order to understand the process by which the host nematode regulates the population growth of *Wolbachia* at a sufficient level to maintain the symbiosis, yet to avoid fitness costs or the pathological consequences of bacterial overgrowth, we investigated the role of autophagy, a conserved intracellular defence mechanism and regulator of cell homeostasis (Voronin *et al.* 2012). Activation of autophagy coincided with the onset of rapid bacterial growth and expansion, which shows that, in spite of their mutualistic association, the nematode's immune system recognises *Wolbachia* as a ‘pathogen’. Genetic and chemical modulation of autophagy activation or suppression resulted in a corresponding decrease or increase in bacterial populations. To test whether drugs which induce the activation of autophagy could lead to a reduction in *Wolbachia* populations *in vivo*, we treated jirds infected with *B. malayi* with rapamycin and spermidine. Treatment with rapamycin or spermidine reduced *Wolbachia* loads by ∼70% for both drugs compared to the control (Voronin *et al.* 2012). These results provide proof-of-concept that drug-induced activation of autophagy is effective at reducing *Wolbachia* populations *in vivo* to the same extent as antibiotic therapy and identifies a novel bactericidal mode-of-action which can be exploited in the discovery and development of new anti-*Wolbachia* treatments.

## A·WOL REGIMEN REFINEMENT

In order to address A·WOL's second goal to optimize regimens of known anti-wolbachial drugs (doxycycline and rifampicin), we carried out a series of phase II field trials with the aim of testing the efficacy of reduced dosage (200 to 100 mg) and to test whether combinations of anti-wolbachial drugs can reduce the treatment period. Two additional studies were initiated to pilot the lead candidate from our registered library screen (minocycline) and to evaluate the efficacy of community-directed doxycycline treatment four years after delivery. (1) A·WOL LF I: RCT phase II trial, doxycycline *vs* doxycycline/rifampicin and doxycycline dose reduction (200 to 100 mg), Ghana. (2) A·WOL oncho I: RCT phase II trial, doxycycline *vs* doxycycline/rifampicin and doxycycline dose reduction (200 to 100 mg), Ghana. (3) A·WOL oncho II: Open label pilot trial, doxycycline *vs* minocycline±albendazole, Ghana. (4) A·WOL oncho III: Evaluation of the effectiveness of community-directed delivery of doxycycline four years after delivery (Cameroon).

All follow-up sampling of phase II and pilot trials is now complete, with ongoing laboratory analysis of primary and secondary endpoints underway, which is expected to be completed by the end of 2013.

In 2007 and 2008, a feasibility trial of community-directed treatment with doxycycline was carried out in two health districts in Cameroon, co-endemic for *O. volvulus* and *L. loa* (Wanji *et al.* 2009). With 17 519 eligible subjects, the therapeutic coverage was 73·8% with 97·5% compliance, encouraging the feasibility of using doxycycline community-directed delivery in restricted populations of this size. The effectiveness of this community-directed delivery of doxycycline was further evaluated four years after delivery (Tamarozzi *et al.* 2012). Statistically significant lower microfilarial prevalence (17·0% [doxycycline plus ivermectin group], 27·0% [ivermectin only group], *P* = 0·014) and load (*P* = 0·012) were found in people that had received doxycycline followed by ivermectin compared to those who received ivermectin only. This study demonstrates the long-term effectiveness of doxycycline treatment delivered with a community-directed strategy even when evaluated four years after delivery in an area of ongoing transmission. This finding shows that a multi-week course of treatment is not a barrier to community-delivery of MDA in restricted populations of this size and supports its implementation to complement existing control strategies for onchocerciasis, where needed (Tamarozzi *et al.* 2012).

## A·WOL MATHEMATICAL MODELLING

An extensive series of trials has shown that doxycycline treatment eliminates *Wolbachia* causing long-term sterilization of adult female filariae and ultimately exerting a macrofilaricidal effect against onchocerciasis and LF. Such trials have been conducted in endemic settings where continual reinfection by drug-naïve worms compromises the evaluation of macrofilaricidal efficacy (Specht *et al.* 2009). This makes it difficult to estimate therapeutic efficacy and compare data from different doxycycline regimens collected at different times post-treatment. A mathematical model was developed which couples the doxycycline-induced depletion of *Wolbachia* from adult *O. volvulus* to the ensuing macrofilaricidal activity (Walker *et al.* unpublished results). The model was fitted to data from clinical trials measuring the *Wolbachia* status and viability of individual female adult worms exposed to a 4-, 5- or 6-week daily dose of 100 or 200 mg oral doxycycline. Doxycycline induces rapid depletion of *Wolbachia*, yet these effects are most apparent 9·5 months after the start of treatment. The estimated therapeutic efficacy of doxycycline in eliminating *Wolbachia* from female *O. volvulus* increases statistically significantly from 92 to 95% from 4 to 5 weeks of treatment and non-significantly from 95 to 97% from 5 to 6 weeks of treatment, irrespective of dose. This model validates the marked macrofilaricidal activity of doxycycline therapy and provides robust statistical support for equivalent efficacy with reduced timeframes and dosage and can be adapted to the analysis of other A·WOL therapies for the treatment of both onchocerciasis and LF.

## ANTI-*WOLBACHIA* TREATMENT IMPROVES CLINICAL DISEASE

Previously, a course of doxycycline was shown not only to possess macrofilaricidal activity, but also lead to significant clinical improvements in the severity of lymphoedema (Debrah *et al.* 2006). In a second trial this outcome was compared with a course of amoxicillin and in patients without active LF infection (Mand *et al.* 2012). Doxycycline-treated patients with lymphoedema (LE) stage 2–3 showed significant reductions in LE severity after 12 and 24 months, regardless of circulating filarial antigen status. Improvement was observed in 43·9% of doxycycline-treated patients, compared with only 3·2 and 5·6% in the amoxicillin and placebo arms, respectively. Both doxycycline and amoxicillin reduced acute dermatolymphangioadenitis attacks. This unexpected outcome showed that improvements in lymphoedema were also found in patients without active infection, which expands the use of this approach as a new and improved tool for morbidity management as part of GPELF (Mand *et al.* 2012).

## CONCLUSIONS

A·WOL has developed a series of validated and robust assays to evaluate drugs and compounds with anti-*Wolbachia* activity which have been used to screen a range of registered, focused and diversity drug libraries to deliver several hundred ‘hits’, which are progressing through the screening funnel with the potential to generate at least one new anti-wolbachial chemotype for eventual deployment as a macrofilaricide. The outcomes of the initial A·WOL programme are progressing through A·WOL II Macrofilaricide Drug Discovery and A·WOL II Macrofilaricide Drug Development programmes.

Regimens of known A·WOL drugs have been optimised for dosage and time-frame to deliver a curative course of treatment equivalent to regimens for prophylaxis for traveller's malaria and acne which can be considered, in restricted populations, to complement existing MDA strategies in ‘hot spot’ foci or residual populations in MDA end-game scenarios, where test and treat strategies become more cost-effective and deliverable than MDA. An example is the decision by Onchocerciasis Elimination Programme for the Americas (OEPA) to use doxycycline in attempts to foreshorten the time to elimination in a focus in Venezuela to achieve the Regional Elimination goal. The WHO Road Map (WHO, 2012) at the time of the commitment to the London Declaration on NTDs has ambitious targets based on existing available preventive chemotherapy tools. However, it is recognized that if there is to be a more rapid drive towards elimination and strategies to tackle the barriers of reduced efficacy of existing drugs and *L. Loa*, A·WOL outcomes should be considered for deployment at the earliest opportunity.
